# Transphyseal Proximal Humeral Aneurysmal Bone Cyst with Pathologic Fracture in a Child: A Case Report

**DOI:** 10.3390/diagnostics16111657

**Published:** 2026-05-28

**Authors:** Taichun Li, Jingmiao Wang, Qin Zhang, Ziming Zhang

**Affiliations:** 1 Department of Pediatric Orthopedics, Shanghai Children’s Hospital, School of Medicine, Shanghai Jiao Tong University, Shanghai 200062, China; litaichun@shchildren.com.cn (T.L.); wangjingmiao@shchildren.com.cn (J.W.); 2 Department of Pediatric Orthopedics, Xinhua Hospital, Shanghai Jiao Tong University School of Medicine, Shanghai 200092, China; selleriezhang@sjtu.edu.cn

**Keywords:** aneurysmal bone cyst, proximal humerus, transphyseal involvement, pathologic fracture, magnetic resonance imaging, pediatric musculoskeletal tumor, case report

## Abstract

**Background and Clinical Significance:** Aneurysmal bone cyst (ABC) is a benign but locally aggressive osteolytic lesion that typically arises in the metaphysis of long bones in children and adolescents. Extension across an open proximal humeral physis into the epiphysis is uncommon and creates a diagnostic challenge because unicameral bone cyst and telangiectatic osteosarcoma may show overlapping radiological features. **Case Presentation:** A 12-year-old girl presented with pain and restricted motion of the left shoulder after a bicycle-related fall. Radiographs demonstrated a large expansile lytic lesion of the proximal humerus with cortical thinning and pathologic fracture. CT showed cortical ballooning, septation-like internal architecture, and extension across the open proximal humeral physis into the epiphysis. MRI demonstrated a multiloculated cystic lesion with thin internal septa and thin peripheral/septal enhancement, without nodular solid enhancement or a bulky extraosseous soft-tissue component. Curettage, bone cement filling, and elastic intramedullary nailing were performed. Histopathology, immunohistochemistry, and *USP6* FISH analysis supported the final integrated diagnosis of ABC, although *USP6* rearrangement was not detected. At 40 months, the patient was pain-free, had essentially normal shoulder motion, had returned to normal school and daily activities, and required no further surgery; radiographs showed stable remodeling without aggressive re-expansion. **Conclusions:** A focused literature review supports that frank transphyseal ABC is rare, particularly in the proximal humerus. The diagnostic contribution of this case lies in the integration of radiography, CT, MRI, histopathology, ancillary testing, and long-term clinical and radiographic follow-up. In a skeletally immature patient with a proximal humeral cystic lesion, transphyseal extension should prompt careful multimodality assessment. Imaging can substantially narrow the differential diagnosis, but integrated radiologic–pathologic confirmation remains essential when malignant mimics are possible.

## 1. Introduction

Aneurysmal bone cyst (ABC) is a benign but locally aggressive osteolytic lesion that usually affects children, adolescents, and young adults. It most often arises in the metaphysis of long bones, but it may also occur in the spine, pelvis, flat bones, and small bones. Contemporary reviews emphasize that ABC is not a single purely reactive entity. Many primary ABCs are associated with *USP6* rearrangements and are now considered clonal neoplastic lesions, whereas secondary ABC-like change can occur in association with other benign or malignant bone tumors. These biological considerations matter clinically because a blood-filled, septated, cystic lesion on imaging does not automatically represent a primary ABC [1,2,3,4].

Radiography remains the first-line examination for a pediatric bone lesion, because it provides information about lesion location, matrix, zone of transition, cortical response, periosteal reaction, and fracture. ABC classically appears as an expansile, geographic, osteolytic lesion with cortical thinning and internal septations. CT is useful for confirming cortical ballooning, the presence or absence of mineralized matrix, and the relationship of the lesion to the cortex, epiphysis, and articular surface. MRI is the most comprehensive modality for local assessment because it depicts cystic architecture, internal septa, enhancement pattern, soft-tissue extension, and the relationship to the physis and epiphysis. Broader pediatric bone-tumor imaging reviews emphasize that lesion location, matrix, margins, periosteal reaction, and extraosseous extension should be integrated before labeling a lesion benign or aggressive [5,6]. Nevertheless, several of these features are not specific, and when fluid–fluid levels are present, they can be seen in ABC, telangiectatic osteosarcoma (TOS), and secondary ABC-like change [1,2,5,7].

The proximal humerus is a common site for pediatric cystic bone lesions, but not all proximal humeral cystic lesions carry the same diagnostic or management implications. Unicameral bone cyst (UBC), also termed simple or solitary bone cyst, is classically a centrally located metaphyseal lesion of the proximal humerus or proximal femur in children and frequently presents with pathologic fracture. UBC may become complex after fracture, and rare cases with extension into the epiphysis have been reported. TOS, by contrast, is an aggressive malignant bone tumor that may be predominantly cystic and may show hemorrhagic cystic areas, including fluid–fluid levels in some cases; therefore, it is the most important malignant mimic of ABC in a child or adolescent with a cystic, expansile long-bone lesion [7,8,9,10,11,12,13]. General pediatric reviews and reports on ABCs with unusual features similarly stress that imaging should be interpreted in a differential diagnostic framework rather than by relying on a single sign [6,14,15].

The present case is diagnostically relevant because the lesion was centered in the proximal humerus, crossed the open proximal humeral physis into the epiphysis, and presented with a pathologic fracture. The physis is often regarded as a relative barrier to tumor spread, so frank transphyseal involvement in a benign cystic lesion is an uncommon and potentially confusing imaging pattern. At the same time, the proximal humeral physis contributes substantially to humeral growth, so physeal crossing creates a reason for long-term surveillance even when the lesion is benign and successfully treated [16,17,18,19,20].

We report a 12-year-old girl with a proximal humeral ABC with transphyseal extension and pathologic fracture. The aim of this case report and focused literature review is to describe the multimodality imaging features that supported ABC, explain the diagnostic distinction from UBC and TOS, and clarify how long-term imaging follow-up is relevant when a lesion traverses the open physis. This case report was prepared in accordance with CARE case-reporting principles, and the episode of care is summarized in a concise timeline.

## 2. Case Presentation

### 2.1. Clinical Presentation and Initial Radiographs

A 12-year-old girl presented with 11 days of pain in the left upper arm and limited shoulder motion after a bicycle-related fall. There was no known history of previous tumor diagnosis, systemic malignancy, constitutional symptoms, or chronic inflammatory disease. Physical examination showed swelling and focal tenderness over the left proximal arm. Active shoulder motion was limited by pain. Distal perfusion, finger motion, and sensation were preserved, and no neurovascular deficit was identified.

Initial anteroposterior and lateral radiographs of the left humerus demonstrated a large expansile geographic lytic lesion centered in the proximal humerus. The lesion produced marked cortical thinning and ballooning and was associated with a pathologic fracture through the proximal humeral metadiaphysis. The proximal extent of the lucent lesion approached the humeral head, and the normal contour of the open proximal humeral physis was indistinct. No obvious mineralized matrix was visible on the radiographs. The combination of an expansile cystic lesion and pathologic fracture raised the possibility of ABC or UBC, but the indistinct physeal margin and apparent extension toward the epiphysis prompted further cross-sectional imaging (Figure 1).

### 2.2. CT and MRI Assessment

CT of the left humerus was performed on a GE Revolution CT system as part of the preoperative diagnostic assessment. The available image annotations indicated acquisition at 100 kV, with thin-section axial images and multiplanar reformations, including a standard axial reconstruction of approximately 0.63 mm and coronal bone-kernel reformations of approximately 1.44 mm. Coronal reformations were used to define the relationship of the lesion to the open proximal humeral physis, epiphysis, and humeral head. CT confirmed a large expansile osteolytic lesion with a thin shell of remaining cortex and internal septation-like architecture. The lesion crossed the open proximal humeral physis and extended into the epiphysis, approaching the subchondral bone of the humeral head. CT did not demonstrate a mineralized osteoid or chondroid matrix. No bulky extraosseous soft-tissue mass was evident. These CT findings supported a cystic expansile lesion rather than a densely mineralized bone-forming tumor, but the transphyseal pattern required cautious interpretation (Figure 2).

MRI was performed to further characterize the lesion, evaluate its internal architecture, and assess the soft tissues. MRI included coronal and axial proton density-weighted fat-suppressed fluid-sensitive sequences, a non-fat-suppressed T1-weighted turbo spin-echo sequence, and axial post-contrast fat-suppressed T1-weighted imaging. These sequences allowed assessment of cystic architecture, internal septa, enhancement pattern, soft-tissue extension, and physeal/epiphyseal involvement. Coronal and axial fluid-sensitive images showed a predominantly cystic and multiloculated lesion with thin internal septa and a thin peripheral rim. The lesion crossed the proximal humeral physis into the epiphysis, confirming the transphyseal pattern suggested by CT. Post-contrast fat-suppressed T1-weighted imaging demonstrated thin peripheral and septal enhancement. There was no nodular solid enhancement, no bulky soft-tissue component, and no imaging evidence of aggressive permeative destruction. The absence of a nodular enhancing solid component was important because TOS remained the principal malignant alternative diagnosis (Figure 3).

The preoperative imaging impression favored ABC, but the case was diagnostically challenging for four reasons: the lesion crossed an open physis, the pathologic fracture could complicate interpretation through hemorrhage and cortical disruption, cystic pediatric bone lesions may overlap on imaging, and TOS needed to be excluded. UBC with pathologic fracture remained a benign differential consideration because of the age of the patient and proximal humeral location. However, the marked expansile remodeling, multiloculated cystic architecture, cortical ballooning, and peripheral/septal enhancement were more supportive of ABC. TOS could not be excluded by imaging alone because it may also appear cystic, but the lack of nodular solid enhancement, bulky extraosseous soft tissue, aggressive periosteal reaction, and mineralized matrix made TOS less likely [7,12,13].

### 2.3. Treatment, Histopathology, and Follow-Up

The patient underwent operative treatment consisting of lesion curettage, tissue sampling, bone cement filling, and retrograde elastic intramedullary nail fixation. The intervention had three goals: to obtain diagnostic tissue, to reduce the cystic lesion burden, and to stabilize the pathologic fracture and weakened proximal humeral segment. Curetted tissue from the lesion wall and internal septation-like components was submitted for intraoperative and permanent pathological assessment. Bone cement was used to fill the treated cavity, and retrograde elastic intramedullary nails were used to provide internal stabilization across the weakened humeral segment.

Immediate postoperative radiographs showed filling of the proximal humeral cavity with bone cement and retrograde elastic intramedullary nail fixation. Serial radiographs were obtained during follow-up. Radiographs from April 2021 through August 2024 demonstrated interval remodeling of the proximal humerus around the cement-filled cavity. On the available images, there was no aggressive re-expansion, humeral head collapse, obvious gross angular deformity, or radiographic finding that suggested active local recurrence. At 40 months after surgery, the patient was pain-free, had essentially normal shoulder motion, had returned to normal school and daily activities, and had not required additional surgery. The treated proximal humerus remained radiographically stable (Figure 4). Because the lesion had crossed the open proximal humeral physis, continued clinical and imaging surveillance until skeletal maturity remains reasonable.

Frozen section examination did not exclude ABC. Permanent histopathologic examination demonstrated blood-filled cystic spaces separated by fibrous septa. Low-power hematoxylin and eosin staining showed hemorrhagic cystic spaces and fibrous septa. Higher-power examination showed fibroblast-like spindle cells, hemosiderin deposition, scattered osteoclast-like multinucleated giant cells, and reactive woven bone within the septa. No definite malignant osteoid production, marked cytologic atypia, or atypical mitotic activity was identified (Figure 5A–D). No definite malignant tumor component or another underlying bone tumor with secondary ABC-like change was identified.

Ancillary immunohistochemical findings included PGM1 positivity, cytokeratin negativity, focal S100 positivity, SATB2 positivity, low Ki-67 proliferative activity, and limited p53 expression. Specifically, one sampled area showed PGM1 positivity, CK negativity, focal S100 positivity, SATB2 positivity, Ki-67 positivity in approximately 5% of cells, and p53 positivity in approximately 10% of cells; another sampled area showed SATB2 positivity and Ki-67 positivity in approximately 1% of cells. FISH analysis using a *USP6* break-apart rearrangement probe did not demonstrate *USP6* rearrangement. Rare separated red/green signal patterns were observed in fewer than 10% of lesional cells, below the laboratory threshold for a positive result (Figure 6). The final diagnosis of ABC was made on the basis of integrated radiological, histopathological, immunohistochemical, molecular, and clinical findings.

The clinical and diagnostic course is summarized in Table 1. The timeline emphasizes the sequence from trauma-related presentation to multimodality imaging, pathological assessment, ancillary testing, treatment, and long-term clinical and radiographic follow-up.

## 3. Discussion

This case illustrates a rare and diagnostically challenging pattern of pediatric ABC: a proximal humeral cystic lesion with pathologic fracture and frank extension across an open physis into the epiphysis. To our knowledge, published reports of transphyseal proximal humeral ABC with pathologic fracture and long-term follow-up are extremely limited. The main value of the case is not simply that ABC occurred in the proximal humerus, which is a recognized site, but that the lesion showed a transphyseal pattern in a skeletally immature patient. This imaging pattern expands the differential diagnosis and increases the need for radiology–pathology correlation. It also changes follow-up priorities because the lesion, the fracture, and the intervention all occurred at or near a growth plate.

The literature provides several relevant points for interpreting this case. First, ABC can be primary or secondary, and the diagnosis should not be made solely by identifying one imaging sign. Second, ABCs adjacent to the physis have long been recognized, but frank transphyseal extension is much less commonly documented. Third, UBC can rarely extend into the epiphysis, especially around the proximal humerus, and therefore may mimic transphyseal ABC in a child with pathologic fracture. Fourth, TOS is the critical malignant mimic because it can be radiographically expansile and cystic on MRI. These points are summarized in the focused literature review in Table 2.

### 3.1. Diagnostic Significance of Transphyseal Involvement

The transphyseal pattern is the most distinctive feature of this case. A cystic lesion of the proximal humerus in a child is often assumed to be metaphyseal, especially if it presents with fracture, but the CT and MRI in this patient demonstrated that the lesion crossed the open physis and reached the epiphysis. This finding matters for two reasons. Diagnostically, it broadens the differential beyond a typical metaphyseal cyst and creates overlap with epiphyseal or transphyseal entities. Prognostically, it raises concern for growth disturbance, because the lesion itself, fracture displacement, curettage, cement, fixation, or postoperative remodeling could affect the proximal humeral physis.

A transphyseal lesion should not automatically be interpreted as malignant. The cases by Arora and colleagues demonstrate that benign ABC can cross an open physis, and the report by Haims and colleagues demonstrates that UBC can also rarely extend into the epiphysis [10,17,18]. However, crossing the physis should prompt more careful imaging review. In the present case, the lesion remained geographic and expansile rather than permeative; the cortex was thinned and ballooned rather than destroyed in a frankly aggressive pattern; and there was no bulky soft-tissue mass. These features supported a benign but locally aggressive process, while the transphyseal extension and fracture justified tissue diagnosis.

The proximal humerus is also a clinically important location because the growth plate remains active during childhood and adolescence. A lesion that crosses this physis may create uncertainty about whether subsequent deformity would be caused by the lesion, the fracture, curettage, cement filling, fixation, or natural remodeling. This is why the imaging report should explicitly state whether a cystic lesion abuts, distorts, or crosses the physis. In the present patient, the follow-up radiographs showed stable remodeling, but the transphyseal involvement remains a reason to continue surveillance until skeletal maturity.

### 3.2. Multimodality Imaging and Differential Diagnosis

The imaging diagnosis in this case depended on pattern recognition across modalities. Radiographs established that the lesion was lytic, expansile, and associated with a pathologic fracture. CT confirmed cortical ballooning, a thin residual cortical shell, internal septation-like architecture, and absence of mineralized matrix. MRI added decisive information by demonstrating a predominantly cystic multiloculated lesion with thin internal septa and thin peripheral/septal enhancement without nodular solid enhancement. These findings collectively favored ABC [1,7].

No single imaging sign was treated as pathognomonic. Although fluid–fluid levels are classically associated with ABC, they are neither required for diagnosis nor specific when present. In this case, the interpretation emphasized lesion margin, cortical behavior, multiloculated cystic architecture, enhancement pattern, and soft-tissue findings. Similarly, physeal crossing was not used as proof of malignancy; it was used as a signal that the case required CT/MRI correlation and histologic confirmation. The teaching point of the present case is that transphyseal extension in a pediatric cystic proximal humeral lesion should trigger careful multimodality assessment and radiologic–pathologic correlation, rather than being interpreted on the basis of rarity alone.

UBC was an appropriate initial differential because the proximal humerus is a classic site for UBC, and fracture is a common presentation. The literature on humeral simple bone cysts emphasizes that fracture risk is clinically important in children [8,9,11]. The difficulty in the present case was that fracture-related hemorrhage can complicate a UBC and can create internal signal heterogeneity. However, UBCs are generally more central and less septated, and the pronounced multiloculation, cortical ballooning, and peripheral/septal enhancement in this case were more consistent with ABC. The prior case of UBC with epiphyseal extension shows that epiphyseal involvement alone does not distinguish ABC from UBC [10].

TOS remained the most important malignant alternative. Studies comparing ABC and TOS emphasize that fluid–fluid levels are not specific. Findings that should increase concern for TOS include an aggressive transition zone, cortical destruction, a bulky soft-tissue mass, mineralized matrix, thick or irregular septa, and nodular enhancing solid tissue [7,12,13]. The present lesion lacked these features. Nevertheless, imaging cannot replace histopathology when TOS is part of the differential diagnosis. The final diagnosis of ABC was therefore supported by imaging and established through integrated histopathological and ancillary assessment.

Table 3 summarizes the core imaging differential diagnosis for a transphyseal cystic proximal humeral lesion in a child, focusing on the four entities most directly relevant to the present diagnostic problem: ABC, UBC, TOS, and secondary ABC-like change. This focused approach emphasizes practical radiologic distinctions among these entities, including lesion matrix, margin, transition zone, periosteal reaction, soft-tissue extension, and enhancement pattern [6,14,15].

For the purposes of preoperative communication, this distinction should be expressed as a probability-based diagnosis rather than as an absolute conclusion. In the present case, the most useful imaging descriptors were the entire pattern: geographic expansion, a thin residual cortical shell, internal septation-like architecture, multiloculated cystic MRI appearance, transphyseal extension, absence of mineralized matrix, absence of bulky extra-osseous soft tissue, and lack of nodular enhancing solid tissue. Stating these positive and negative features in the report can help the surgical team plan biopsy, stabilization, and follow-up while preserving awareness of malignant mimics.

Histopathologic and ancillary findings were important because imaging alone cannot definitively separate ABC from malignant mimics or from secondary ABC-like change in another tumor. In this case, permanent histopathologic examination showed blood-filled cystic spaces separated by fibrous septa, with fibroblast-like spindle cells, osteoclast-like multinucleated giant cells, hemosiderin deposition, and reactive woven bone (Figure 5). Immunohistochemical testing showed SATB2 positivity with low Ki-67 proliferative activity and no cytokeratin expression, supporting an osteogenic/reactive lesion rather than epithelial malignancy. *USP6* break-apart FISH did not demonstrate *USP6* rearrangement; rare separated red/green signal patterns were below the laboratory threshold for a positive result (Figure 6). Although *USP6* rearrangement is a useful molecular feature in many primary ABCs, a negative *USP6* FISH result does not exclude ABC when the radiologic and histologic findings are otherwise supportive. Therefore, the diagnosis in the present patient was based on integrated radiologic–pathologic correlation rather than on a single molecular result [1,2,4].

### 3.3. Pathologic Fracture, Treatment Choice, and Follow-Up

Pathologic fracture was an important part of the presentation. A fracture can be the first event that brings a cystic bone lesion to clinical attention, and it can also alter imaging appearances through hemorrhage, fluid levels, edema, and cortical disruption. In pediatric bone tumors and tumor-like lesions, pathologic fracture is not by itself proof of either benignity or malignancy; instead, it must be interpreted with lesion morphology, patient age, location, matrix, soft-tissue findings, and enhancement pattern [23,24]. In this patient, the fracture increased the urgency of stabilization and made UBC a reasonable differential consideration, but the broader imaging pattern still favored ABC.

The treatment strategy combined curettage, bone cement filling, and elastic intramedullary nail fixation. The purpose was both diagnostic/therapeutic and mechanical: to remove and sample the lesion, fill the large cavity, and stabilize the pathologic fracture. Current reviews describe several treatment options for ABC, including curettage with or without adjuvants, bone grafting or cementation, percutaneous sclerotherapy, image-guided approaches, embolization, denosumab in selected contexts, and en bloc resection in anatomically appropriate or aggressive cases [25,26,27,28,29,30,31,32,33]. For a proximal humeral lesion with fracture and cortical weakening, the surgical approach used here was clinically reasonable and allowed histologic confirmation.

This case should not be read as evidence that cement filling and elastic nailing are superior to less invasive strategies for all pediatric ABCs. Rather, it shows how treatment selection was linked to the presenting circumstances: the lesion was large, the cortex was markedly thinned, a pathologic fracture was present, and tissue diagnosis was needed because of malignant mimics. For lesions without fracture or mechanical compromise, image-guided sclerotherapy or other minimally invasive approaches may be considered. The literature review therefore supports individualized management rather than a single preferred method.

Local recurrence is a recognized concern after ABC treatment, particularly in children and in lesions close to the growth plate. The literature does not support a single universal treatment algorithm for all ABCs, and recurrence risk is influenced by age, site, lesion extent, treatment method, and completeness of local control [25,26,27,28,29,30,31,32,34]. The available 40-month follow-up in this patient showed remodeling around the cement-filled cavity without aggressive re-expansion, humeral head collapse, or gross angular deformity on radiographs. Clinically, the patient was pain-free, had essentially normal shoulder motion, had returned to normal school and daily activities, and had not required additional surgery. These findings are reassuring, but they do not eliminate the need for continued surveillance because the patient was skeletally immature at diagnosis.

Growth-plate surveillance is particularly relevant in this case. Imaging reviews of pediatric growth plate disturbances show that partial physeal arrest, physeal bars, angular deformity, and limb-length discrepancy may not be immediately apparent after injury or intervention [20]. Pastor and colleagues recently emphasized the need to monitor ABCs abutting the growth plate [19]. Our case adds to that discussion because the lesion did not merely abut the physis; it crossed the open proximal humeral physis into the epiphysis. Continued follow-up until skeletal maturity is therefore prudent even after radiographic stability has been achieved.

### 3.4. Limitations

This report has limitations. It describes a single patient and therefore cannot estimate the frequency, recurrence risk, or growth-related consequences of transphyseal proximal humeral ABC. Although the patient was pain-free, had essentially normal shoulder motion, returned to normal school and daily activities, and required no additional surgery at 40 months, formal goniometric range-of-motion measurements, patient-reported outcome scores, quantitative limb-length measurements, and dedicated imaging for a physeal bar were not available. Detailed numerical MRI acquisition parameters, including TR/TE, slice thickness, field of view, matrix, and flip angle, were not available from the exported image set. Representative hematoxylin-and-eosin and *USP6* FISH images support radiologic–pathologic correlation; however, detailed digital whole-slide pathology review and broader molecular testing beyond *USP6* break-apart FISH were not available. In addition, *USP6* rearrangement was not detected, so molecular confirmation of a *USP6*-rearranged primary ABC was not available.

### 3.5. Practical Diagnostic Message

A cystic proximal humeral lesion crossing an open physis in a child should be reported systematically. Radiographs should establish whether the lesion is geographic or aggressive and whether fracture, matrix, periosteal reaction, or soft-tissue abnormality is present. CT should clarify the residual osseous shell, cortical integrity, and relationship to the physis and epiphysis. MRI should focus on multiloculation, internal septa, enhancement pattern, soft-tissue extension, and the presence or absence of nodular solid tissue.

In the present patient, the most useful diagnostic information was the entire pattern rather than a single sign: marked expansion, multiloculation, thin septal/peripheral enhancement, transphyseal extension, and absence of a nodular solid component or bulky soft-tissue mass. This pattern favored ABC, but histopathologic assessment and ancillary testing remained necessary because TOS and secondary ABC-like change can overlap with ABC on imaging. The case therefore provides a focused diagnostic example for radiologists, orthopedic surgeons, and pediatric clinicians who evaluate cystic bone lesions near the growth plate.

## 4. Conclusions

Transphyseal involvement by ABC in the proximal humerus of a skeletally immature patient is uncommon and can be confused with UBC, TOS, or secondary ABC-like change in another tumor. In this case, radiographs, CT, and MRI showed an expansile multiloculated lesion with cortical ballooning, thin internal septa, thin septal/peripheral enhancement, and no nodular solid component or bulky soft-tissue mass. These features favored ABC, and the final diagnosis was established through integrated radiologic, histopathologic, immunohistochemical, molecular, and clinical assessment.

The main diagnostic lesson is that a transphyseal pediatric cystic bone lesion requires integration of radiographic morphology, CT anatomy, MRI enhancement pattern, histopathology, ancillary testing, and follow-up rather than reliance on any single imaging feature. In this patient, the available 40-month clinical and radiographic follow-up showed no pain, essentially normal shoulder motion, return to normal school and daily activities, no need for additional surgery, and stable postoperative remodeling without aggressive re-expansion. Because the lesion crossed the proximal humeral physis, continued surveillance until skeletal maturity remains appropriate even after stable postoperative remodeling.

## Figures and Tables

**Figure 1:**
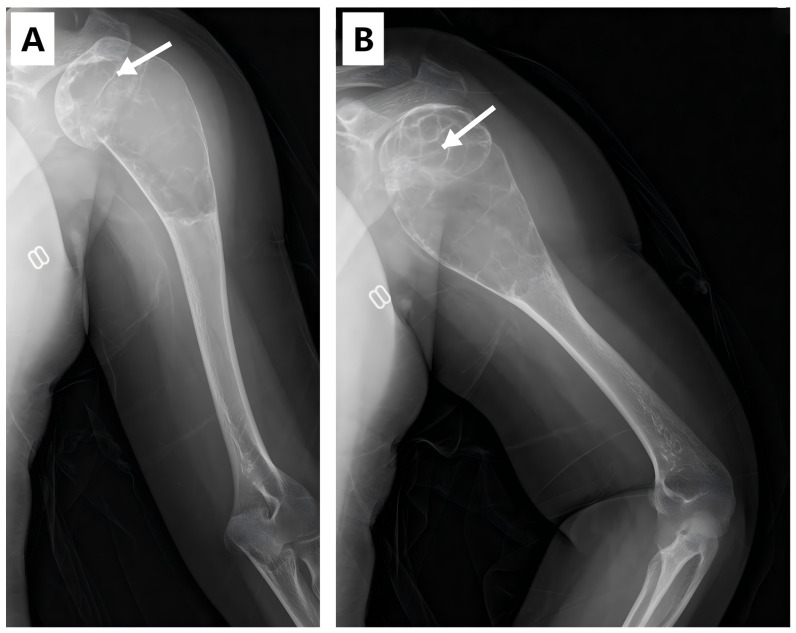
Initial radiographs of the left humerus. (**A**) Anteroposterior and (**B**) lateral radiographs demonstrate a large expansile geographic lytic lesion of the proximal humerus with marked cortical thinning and a pathologic fracture through the proximal humeral metadiaphysis. The lesion extends proximally toward the humeral head. White arrows indicate the proximal humeral physeal region, where the normal radiographic contour of the open physis is partially obscured by the lesion, raising suspicion for transphyseal involvement.

**Figure 2:**
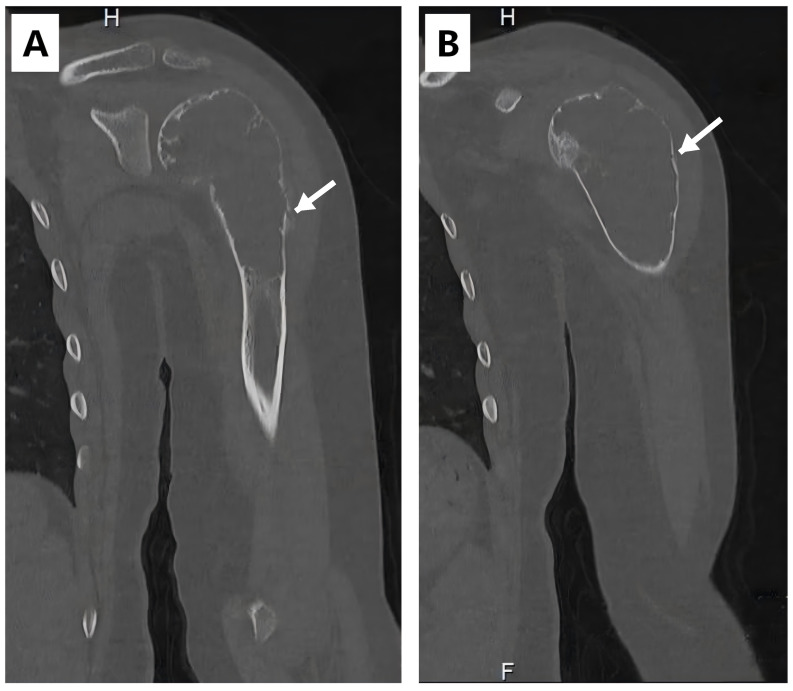
Coronal CT reformations of the left proximal humerus. (**A**,**B**) Coronal CT images demonstrate a large expansile osteolytic lesion with marked cortical thinning, a thin expanded osseous shell, and internal septation-like architecture. The lesion extends across the open proximal humeral physis into the epiphysis and approaches the subchondral bone. White arrows indicate the pathologic fracture through the weakened proximal humeral metadiaphysis.

**Figure 3:**
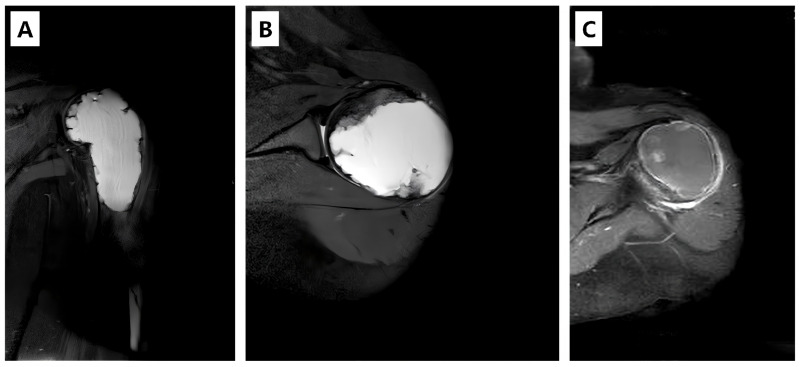
MRI of the left proximal humeral lesion. (**A**) Coronal and (**B**) axial fluid-sensitive MR images show a predominantly cystic, multiloculated lesion extending across the proximal humeral physis into the epiphysis, with thin internal septa and a thin peripheral rim. (**C**) Axial post-contrast fat-suppressed T1-weighted MR image shows thin peripheral and septal enhancement without nodular solid enhancement or a bulky extraosseous soft-tissue component.

**Figure 4:**
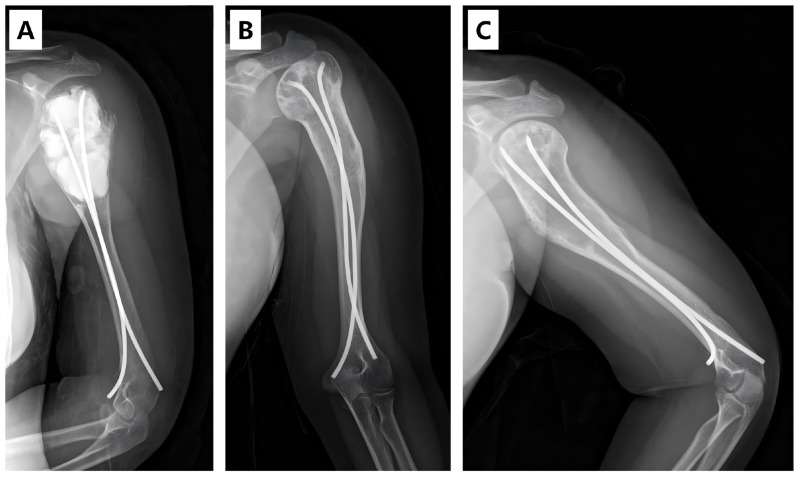
Postoperative and 40-month follow-up radiographs of the left humerus. (**A**) Immediate postoperative radiograph shows bone cement filling of the treated cavity and retrograde elastic intramedullary nail fixation. (**B**,**C**) Forty-month follow-up anteroposterior and lateral radiographs show remodeling and sclerosis of the treated proximal humeral lesion, without aggressive re-expansion, humeral head collapse, or gross angular deformity.

**Figure 5:**
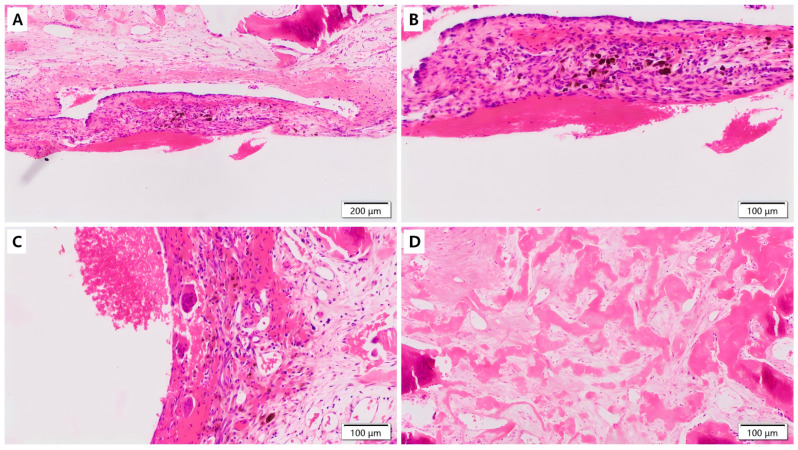
Histopathological findings of the lesion. (**A**–**D**) are representative hematoxylin and eosin-stained fields from the same surgical specimen. (**A**) Low-power hematoxylin and eosin-stained section shows blood-filled cystic spaces separated by fibrous septa (scale bar = 200 μm). (**B**) Higher-power hematoxylin and eosin-stained section shows fibroblast-like spindle cells and hemosiderin deposition within the fibrous septa (scale bar = 100 μm). (**C**) Higher-power hematoxylin and eosin-stained section shows osteoclast-like multinucleated giant cells within the lesion (scale bar = 100 μm). (**D**) Higher-power hematoxylin and eosin-stained section shows reactive woven bone within the fibrous septa (scale bar = 100 μm). No definite malignant osteoid production, marked cytologic atypia, or atypical mitotic activity was identified.

**Figure 6:**
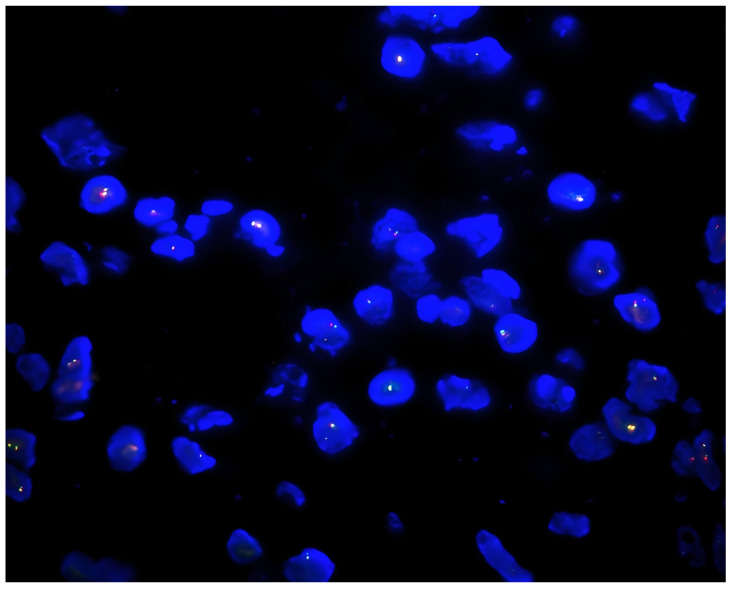
*USP6* break-apart fluorescence in situ hybridization (FISH) analysis of the lesion. Lesional cell nuclei are counterstained with DAPI. Most nuclei show fused or closely apposed red and green signals. Although rare separated red/green signal patterns were observed, they were present in fewer than 10% of lesional cells, below the laboratory threshold for a positive result. The assay was therefore interpreted as negative for *USP6* rearrangement.

**Table 1:** Clinical timeline of the episode of care.

Time Point	Event	Key Diagnostic or Clinical Information
11 days before presentation	Bicycle-related fall	Left upper-arm pain and limited shoulder motion developed after trauma.
Initial presentation	Clinical examination and radiographs	Swelling and tenderness were present over the left proximal arm; distal neurovascular status was preserved. Radiographs showed a large expansile proximal humeral lytic lesion with cortical thinning and pathologic fracture.
Preoperative diagnostic assessment	CT and MRI	CT demonstrated cortical ballooning, a thin residual osseous shell, septation-like architecture, and transphyseal extension into the epiphysis. MRI showed a multiloculated cystic lesion with thin internal septa and thin peripheral/septal enhancement, without nodular solid enhancement or bulky soft-tissue mass.
Operative treatment	Curettage, cement filling, and fixation	Tissue was obtained for pathological assessment. The lesion cavity was treated with curettage and bone cement filling, and the pathologic fracture/weak humeral segment was stabilized using retrograde elastic intramedullary nails.
Pathological and ancillary assessment	Histology, immunohistochemistry, and *USP6* FISH	Histology showed blood-filled cystic spaces separated by fibrous septa with fibroblast-like spindle cells, hemosiderin deposition, osteoclast-like multinucleated giant cells, and reactive woven bone. Immunohistochemistry and *USP6* FISH were incorporated into the final integrated diagnosis; *USP6* rearrangement was not detected.
April 2021–August 2024	Serial follow-up radiographs	Radiographs showed interval remodeling around the cement-filled cavity without aggressive re-expansion, humeral head collapse, gross angular deformity, or imaging findings of active local recurrence on the available images.
40 months after surgery	Latest available clinical and radiographic follow-up	The patient was pain-free, had essentially normal shoulder motion, had returned to normal school and daily activities, and did not require additional surgery. Radiographs showed stable remodeling of the treated proximal humerus. Because the lesion crossed the proximal humeral physis, continued clinical and imaging surveillance until skeletal maturity remains appropriate.

**Table 2:** Focused literature review relevant to the present case.

Reference	Study Type/Source	Key Literature Finding	Relevance to the Present Case	Contribution of the Present Case
Restrepo et al., 2022 [1]	Narrative review of ABC pathophysiology, histology, imaging and treatment	ABCs commonly present as expansile lytic lesions; MRI may show fluid–fluid levels, septations, enhancement pattern, physeal/epiphyseal extension and soft-tissue involvement.	Provides the general multimodality diagnostic framework.	Applies the framework to an unusual proximal humeral lesion crossing an open physis with pathologic fracture.
Nasri and Reith, 2023 [2]	Pathology-focused review	Primary ABC may be neoplastic, whereas secondary ABC-like change can occur in other tumors; histology typically shows blood-filled spaces and fibrous septa with giant cells and reactive bone.	Supports radiology–pathology correlation rather than reliance on fluid–fluid levels alone.	Adds imaging–pathology correlation in a pediatric transphyseal proximal humeral lesion.
Capanna et al., 1985 [16]	Classic juxtaepiphyseal ABC report/series	ABCs can occur adjacent to the physis and require attention to growth-plate involvement.	Provides historical context for physeal proximity as a meaningful imaging feature.	The current case was not only juxtaepiphyseal but frankly transphyseal with epiphyseal extension.
Arora et al., 2011 [17]	Case report of proximal tibial ABC crossing an open physis	A benign ABC can rarely cross an open physis despite the common concept of the physis as a barrier.	Direct precedent for transphyseal ABC.	Extends the transphyseal ABC literature to the proximal humerus.
Arora et al., 2021 [18]	Case report of distal tibial ABC crossing an open physis	Multimodality imaging and pathology confirmed an ABC involving metaphysis, physis and epiphysis.	Provides another direct precedent for transphyseal ABC.	Adds a different anatomic site, fracture context, cement filling/fixation and 40-month follow-up.
Pastor et al., 2025 [19]	Pediatric study of ABCs abutting the growth plate	Growth-plate proximity is relevant for treatment planning and follow-up.	Supports continued surveillance when ABC is near or involves the physis.	Adds frank transphyseal involvement rather than simple abutment.
Haims et al., 1997 [10]	Case report of proximal humeral UBC with epiphyseal extension	UBC can rarely extend through the physis into the epiphysis.	Shows that UBC is a realistic benign differential diagnosis.	In this case, multiloculation and enhancement pattern favored ABC over UBC.
Sivakumar et al., 2022 [21]	Pediatric proximal humeral ABC case	Proximal humeral ABC can occur in children and may require repeated treatment and follow-up.	Confirms relevance of the proximal humerus as a pediatric ABC site.	Adds transphyseal extension and long-term radiographic stability after surgical treatment.
Singh et al., 2025 [22]	Proximal humeral ABC mimicking TOS	Aggressive ABC in the proximal humerus can mimic TOS; biopsy and, when available, molecular testing can clarify diagnosis.	Highlights the danger of assigning hemorrhagic cystic imaging features to ABC without considering malignancy.	Illustrates imaging features favoring ABC while still relying on histology.
Zishan et al., 2020 [7]	Retrospective imaging comparison of ABC and TOS	ABC is favored by expanded cortex, lack of soft-tissue mass, predominantly fluid-filled lesion and thin septal enhancement; TOS more often has aggressive destruction and thick/nodular enhancement.	Provides key criteria for ABC-versus-TOS differentiation.	Demonstrates these criteria in a child with proximal humeral pathologic fracture.
Cederberg et al., 2023 [5]	COG/SPR white paper on pediatric bone tumor imaging	Pediatric bone tumor work-up begins with radiographs and uses MRI/CT and biopsy according to diagnostic need.	Supports the stepwise radiograph-CT-MRI-pathology workflow.	Provides a practical example of multimodality imaging narrowing the differential diagnosis before histology.
Di Costa et al., 2024 [23]	Systematic review of pathological fractures in ABC	Pathologic fracture is a clinically relevant presentation of ABC and influences stabilization and follow-up decisions.	Supports the importance of addressing fracture and lesion in the same diagnostic pathway.	Adds a transphyseal proximal humeral case treated with cement filling and elastic intramedullary fixation.

Abbreviations: ABC, aneurysmal bone cyst; CT, computed tomography; MRI, magnetic resonance imaging; TOS, telangiectatic osteosarcoma; UBC, unicameral bone cyst.

**Table 3:** Simplified imaging differential diagnosis of a transphyseal cystic proximal humeral lesion in a child.

Entity	Key Imaging Clues	How the Present Case Compared	Diagnostic Implication
Primary ABC	Expansile geographic lytic lesion with cortical ballooning, thin osseous shell, internal septations, multiloculated cystic architecture, and thin peripheral/septal enhancement; fluid–fluid levels may be present but are not required.	Strong fit: expansile remodeling, septation-like architecture, multiloculated cystic appearance, thin enhancement, and no nodular solid component. Histology and ancillary testing supported the final integrated diagnosis.	Most likely diagnosis, but tissue confirmation remains necessary when malignant mimics are possible.
UBC/simple bone cyst with fracture or rare epiphyseal extension	Usually central metaphyseal proximal humeral lesion; may fracture and become hemorrhagic, but is often less expansile and less multiloculated than ABC.	Considered because of the age, proximal humeral site, and fracture; less favored because of marked multiloculation, cortical ballooning, and septal/peripheral enhancement.	Important benign differential, particularly after fracture; atypical imaging requires biopsy or histology.
Telangiectatic osteosarcoma	Cystic malignant mimic that may show fluid–fluid levels; concern increases with aggressive destruction, wide transition zone, mineralized matrix, bulky soft-tissue mass, or nodular enhancing solid tissue.	Less favored because the lesion lacked nodular solid enhancement, bulky extraosseous soft tissue, mineralized matrix, and aggressive permeative destruction.	Critical malignant mimic; must be excluded when imaging is not fully typical of a benign ABC.
Secondary ABC-like change in another tumor	Fluid–fluid levels can occur within another benign or malignant bone tumor; imaging may reveal a solid component, matrix, unusual epicenter, or aggressive features of the underlying lesion.	No imaging or histologic evidence of another underlying bone tumor was identified in the available material.	Radiologic–pathologic correlation is essential before labeling a cystic hemorrhagic lesion as primary ABC.

Abbreviations: ABC, aneurysmal bone cyst; CT, computed tomography; MRI, magnetic resonance imaging; TOS, telangiectatic osteosarcoma; UBC, unicameral bone cyst.

## Data Availability

Data sharing is not applicable to this article because no datasets were generated or analyzed beyond the presented clinical case.

## Abbreviations

The following abbreviations are used in this manuscript:

ABC	aneurysmal bone cyst
CT	computed tomography
MRI	magnetic resonance imaging
FISH	fluorescence in situ hybridization
IHC	immunohistochemistry
TOS	telangiectatic osteosarcoma
UBC	unicameral bone cyst
USP6	ubiquitin-specific peptidase 6
